# Supplementary material for: Comparative multiomics analysis of cell physiological state after culture in a basket bioreactor
Source: Sci Rep. 2022 Nov 23;12:20161. doi: 10.1038/s41598-022-24687-4 (PMC9686226; doi:10.1038/s41598-022-24687-4)
Supplement: Supplementary file 1 — Supplementary Information 1. [file 41598_2022_24687_MOESM1_ESM.zip › raw data/Transcriptomic raw data/6.Enrichment/2.KEGG/TE1vsTC1/ALL/src/csab05133.html]

csab05133


- csab:103241278

- Down regulated genes

103241278(-4.29901042433954)

- csab:103226390

- Up regulated genes

103226390(2.68260263702081)

- csab:103241278

- Down regulated genes

103241278(-4.29901042433954)

- csab:103221732

- Down regulated genes

103221732(-2.06930923500636)

- csab:103241278

- Down regulated genes

103241278(-4.29901042433954)

Close
